# Point-of-Care Lung Ultrasound in Small Animal Emergency and Critical Care Medicine: A Clinical Review

**DOI:** 10.3390/ani15010106

**Published:** 2025-01-05

**Authors:** Andrea Armenise

**Affiliations:** Freelance Veterinarian, 70124 Bari, Italy; andreaarmenisevet@gmail.com

**Keywords:** canine lung ultrasound, feline lung ultrasound, canine thoracic POCUS, feline thoracic POCUS, respiratory distress ultrasonography

## Abstract

Thoracic point-of-care ultrasound (T-POCUS) has grown in popularity and usage in small animal emergencies and critical care settings due to its non-invasive nature, mobility, and ability to acquire images in real time. This review summarizes current understanding about T-POCUS in dogs and cats with respiratory illnesses, including normal thoracic ultrasonography appearance and numerous pathological situations. The basics of T-POCUS are covered, including equipment, scanning procedures, and picture settings. Practical applications in patients with respiratory distress are discussed, with an emphasis on pleural space abnormalities and lung diseases. Ultrasound results define pulmonary disorders such as pneumonia, atelectasis, cardiogenic and non-cardiogenic pulmonary edema, lung lobe torsion, pulmonary fibrosis, pulmonary thromboembolism, pulmonary neoplasms, and pulmonary bleeding. The evaluation focuses on T-POCUS diagnostic skills in a variety of clinical settings. Limitations and the need for more study to standardize techniques, establish agreed terminology, and create specialized educational routes are highlighted.

## Simple Summary

Thoracic point-of-care ultrasound (T-POCUS) is an important tool for examining pulmonary disorders and has been used to diagnose a wide range of canine and feline respiratory problems. Its non-invasive nature, portability, and ability to produce real-time images make it ideal for veterinary practice. Scanning procedures, probe selection, and picture settings all contribute to accurate interpretation. T-POCUS can be used to diagnose a variety of pleural and pulmonary problems, including pneumothorax, pleural effusion, pneumonia, atelectasis, cardiogenic and non-cardiogenic pulmonary edema, lung lobe torsion, pulmonary fibrosis, pulmonary hemorrhage, pulmonary thromboembolism, and malignancies. B-lines, shred sign, and tissue-like sign are key ultrasound findings that help in diagnosis. T-POCUS is also useful for evaluating illness development and response to therapies. Despite its strengths, further studies are needed to establish its diagnostic accuracy, as well as standardized protocols and proper terminology.

## 1. Introduction

In 2008, the first article on the use of thoracic ultrasound for evaluating canine trauma patients was published [1]. Since then, interest and adoption by researchers and clinicians have steadily increased, leading to its integration into the array of diagnostic tools available today for managing patients with respiratory distress.

The non-invasiveness of ultrasound, along with the portability of equipment that enables point-of-care evaluations in hospital wards, emergency rooms, and operating rooms has facilitated its widespread adoption. Additionally, the gradual reduction in acquisition costs has promoted its global use in small animal emergency and critical care settings.

The accurate interpretation of artifacts generated by the interaction between ultrasound waves and the pleuropulmonary or air interface has allowed for the characterization of various pathological aspects. In some clinical scenarios, ultrasound has demonstrated greater sensitivity and specificity than traditional radiography, a finding already established in human medicine [1,2,3,4].

This method depends not only on the technical characteristics of the ultrasound machine and, to some extent, on scanning technique but, most critically, on the operator’s knowledge and expertise, which are essential for real-time image visualization and interpretation.

The purpose of this review is to present the current knowledge on thoracic point-of-care ultrasound (T-POCUS) in small animal patients with respiratory diseases, covering normal thoracic ultrasound appearance and examining different sonographic findings across various pathological respiratory conditions in dogs and cats. Additionally, the review highlights limitations and future perspectives for clinical applications.

## 2. Fundamentals of T-POCUS

Ultrasound machines generate and process ultrasound waves, converting them into images displayed on the screen. This brief summary, however, involves a complex series of physical principles underlying image generation. For a more in-depth explanation of these principles, readers are referred to the broader scientific literature [5].

Before discussing T-POCUS findings under normal conditions, it is essential to outline the steps for performing the examination.

The ideal position for the exam is sternal or standing, allowing evaluation of both hemithoraces without any distortion due to recumbency. Fur clipping is not generally necessary [6]; however, in some cases, alcohol and ultrasound gel alone are insufficient, so localized clipping may be beneficial [2].

The most commonly used probe for small animal ultrasound is the variable-frequency microconvex, as it is versatile and provides high-quality images across a range of canine sizes and is also suitable for cats. Its concave shape allows it to fit comfortably between the intercostal spaces, minimizing patient discomfort while providing high-quality imaging.

The probe should be placed in the intercostal space in a longitudinal position, perpendicular to the sagittal plane, with the probe marker oriented cranially. The marker, indicated by a symbol that varies by manufacturer, is a guide for proper orientation and appears on the left side of the screen. During evaluation, the probe should be tilted as widely as possible, making small rotations along its main axis and tilting the tail of the probe to direct the ultrasound beam not only perpendicularly to the sagittal plane but also at oblique angles to avoid missing any areas.

To better characterize the extent of certain pathologies and/or define the boundaries, avoiding artifacts produced by bones, it can be useful to switch from a longitudinal scan to a transverse one by rotating the probe 90 degrees to the right of the patient, as is commonly conducted in ultrasound [6].

High-frequency linear probes offer better definition of superficial structures and are useful when the pleuropulmonary interface is irregular, necessitating enhanced image quality for accurate interpretation.

Both linear and microconvex probes yield similar diagnostic results for complex respiratory conditions [7]; the primary difference lies in their size. While the concave shape of the microconvex probe allows it to fit easily between intercostal spaces, the linear probe can simultaneously visualize 3–4 intercostal spaces in small patients and cats, providing a useful overall view that may be difficult to achieve with bulkier probes in some cases.

The advent of handheld portable ultrasound devices has further improved usability, facilitating rapid image acquisition and enhancing portability.

However, certain limitations remain, and an ideal machine for all scenarios has yet to be developed [8]. Given the pace of technological evolution, more advanced devices will likely emerge over the next decade.

**Figure 1:**
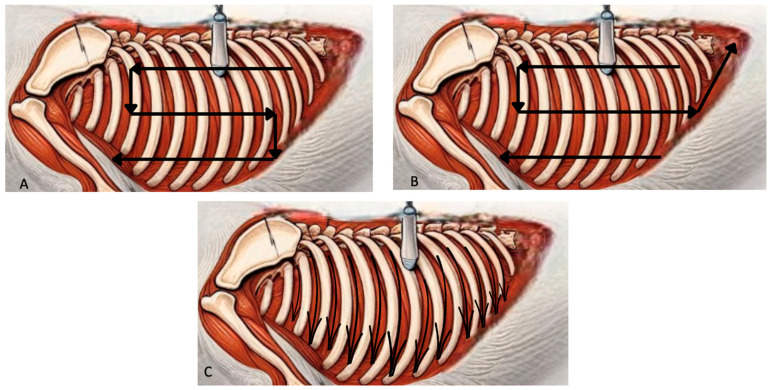
Schematic representation of T-POCUS scanning protocols: (**A**) Armenise’s S-shaped (the arrows show where to position the probe and how to proceed with the scans. Starding from the caudal dorsal position and advance cranially, then direct the probe to the middle region of the thorax in a cranio-caudal direction, and lastly directing the probe ventrally in a caudo-cranial way); (**B**) the Calgary approach (starting at the center caudal point, the probe continues to slide dorsocaudally along the diaphragmatic contour. The remaining evaluation is the same as that of the Armenise’s S-shaped); (**C**) Vezzosi’s protocol (all intercostal spaces are scanned by placing the probe dorsally and directing it ventrally, either cranio-caudally or caudo-cranially).

In human medicine, it is well established that scanning techniques covering the largest possible thoracic surface are preferable, as they provide a more comprehensive description of present pathologies [9]. Although the relevant veterinary literature is limited [10,11,12], extended scanning techniques, such as the modified Vezzosi [13] and Armenise [2] protocols or the Calgary protocol [14], are commonly used in recent studies. For detailed descriptions of these methods, readers are directed to original publications (Figure 1).

The subxiphoid scan, also used for thoracic evaluation [1,15], should be performed with caution, particularly in cases of suspected high intra-abdominal pressure.

It is important to note that the absence of standardized thoracic scanning techniques in veterinary medicine has led to variability in results, which often contradict findings established in human medicine.

Proper image settings are the next step after probe contact with the thoracic wall. Adjustments should balance time gain compensation and gain to produce a well-contrasted, hyperechoic pleural line distinct from the superficial tissue layers, with a depth setting of approximately 5 cm and the focal point at or slightly below the pleural line to better define its motion.

**Figure 2:**
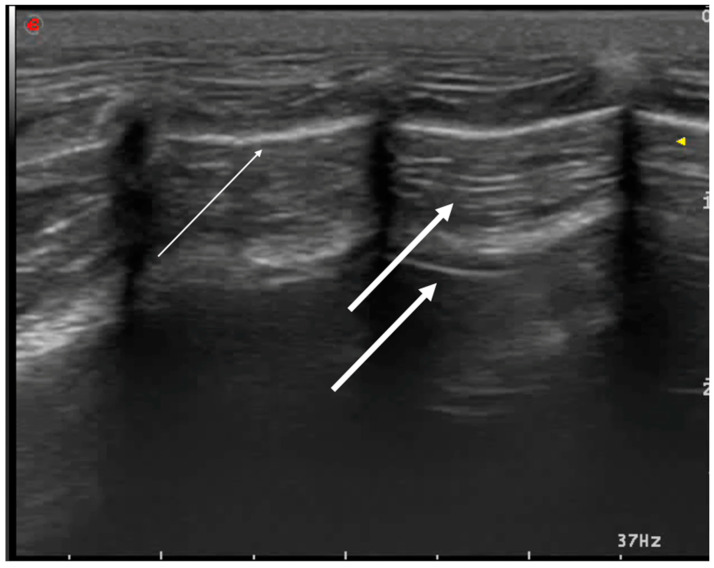
Pleural normal appearance (thin white arrow) with A-lines (thick white arrows).

The initial focus should be on whether the hyperechoic pleural line moves. During respiration, the parietal pleura remains attached to the chest wall, while the visceral pleura, attached to the lung, moves with it, creating a sliding image that synchronizes with respiratory movements—known as the sliding sign. The hyperechoic pleural line typically appears thin and more clearly defined with a linear probe. Due to the presence of air in the lungs and the acoustic impedance difference between air and tissues, ultrasound waves are reflected back as parallel, equidistant hyperechoic lines originating from the pleural line and extending to the bottom of the image. These reverberation artifacts are known as A-lines [16] (Figure 2).

In certain cases, the sliding movement appears as a flickering motion called the lung pulse. This occurs in healthy patients between respiratory phases, when heartbeats propagate through the superficial lung, causing this sign to appear [17]. It is particularly common in cats, according to personal experience.

B-lines are reverberation vertical artifacts that assume critical importance due to their association with increased lung density and reduced air content, generated by subpleural fluid, exudate, blood, collagen, or cellular infiltration [18]. The most recent theory, known as the acoustic trap, suggests that these artifacts are caused by a small volume of fluid surrounded by aerated alveoli, with an acoustic channel above the trap at the pleural line. When an ultrasound beam enters this trap through the channels, it becomes trapped and reflects multiple times off the walls of the aerated alveoli [18]. As the pathology worsens, the number of acoustic traps and B-lines increases. Eventually, the B-lines become so dense that they merge, forming a coalescent appearance [19].

**Figure 3:**
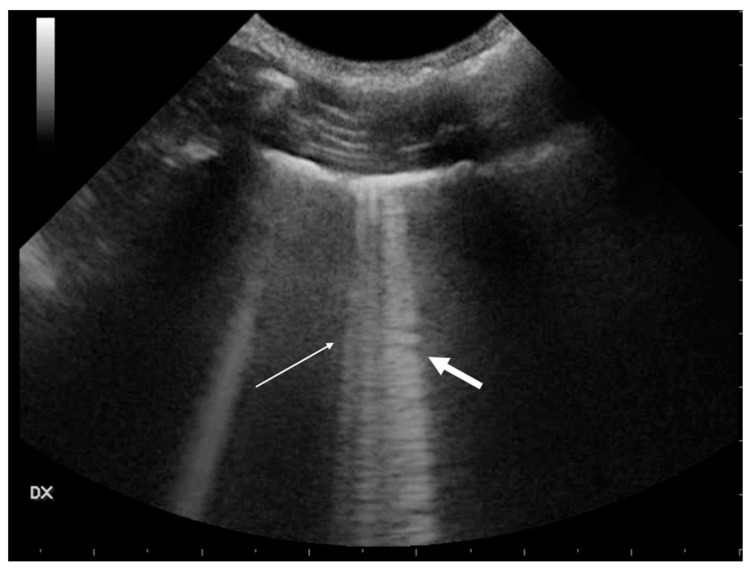
B-lines. The thin white arrow indicates the typical aspect of a B-line; the thick white arrow indicates coalescent B-lines.

B-lines appear as fine, laser-like vertical lines extending from the pleural line to the bottom of the screen without attenuation, widening as they extend downward [20] (Figure 3).

In both human and small animal patients, a hemithorax is considered normal if it contains no more than 3 B-lines [9,10,21,22].

Various other vertical artifacts have been described, though they generally lack clinical significance and are described in greater detail elsewhere [22].

**Figure 4:**
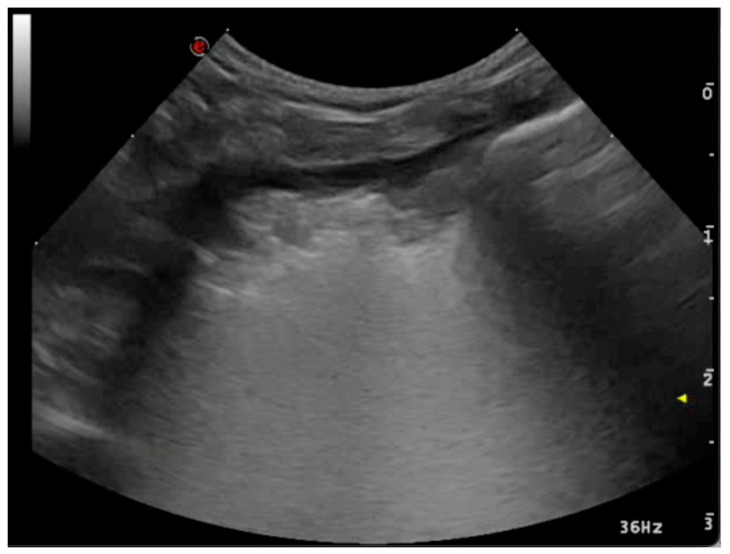
C-lines. Vertical artifacts generated within a consolidated region of the lung.

**Figure 5:**
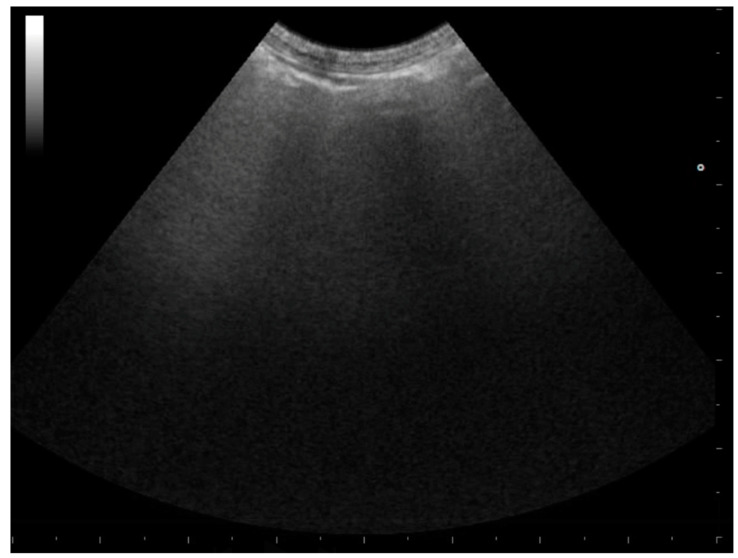
E-lines. Subcutaneous vertical artifacts that cover all the deeper structures.

The only two notable vertical artifacts are the C-lines and the E-lines. C-lines are vertical artifacts generated by the geometric transformation of air gaps at the tissue–air interface or inside portions of a consolidated lung [23] (Figure 4); E-lines arise from the subcutaneous space and extend to the bottom of the image, generated by subcutaneous emphysema [24]. E-lines are stationary, not moving with respiration and obscure the pleural line and any lung signs, whether normal (such as A-lines) or abnormal (pleural effusions, consolidations) [24] (Figure 5).

## 3. Practical Applications in the Patient with Respiratory Distress

The various ultrasound characteristics associated with different respiratory conditions are closely related to the underlying causes and the body’s response to the pathological insult. These characteristics vary depending on the affected anatomical region. Below, we detail these findings, dividing them into pleural space disorders, lung pathologies, thoracic disorders, and a miscellany of ultrasound signs.

### 3.1. Pleural Space Disorders

Pleural space disorders include the accumulation of air or fluid between the pleural layers and primary pleural diseases.

#### 3.1.1. Pneumothorax

As previously discussed, air prevents the propagation of ultrasound waves, allowing visualization only of the fixed parietal pleura, which will appear motionless despite the patient’s respiratory movements, often accompanied by rapid or labored breathing [9].

When pneumothorax is suspected, it is beneficial to reduce the ultrasound gain and tilt the probe tail upward, directing the ultrasound beam obliquely downward. Performing the scan in a dimly lit environment aids in clearly visualizing the absence of the sliding sign, as the patient’s accelerated respiratory pattern can complicate interpretation. Additionally, to optimize the search, the dorsal regions should be examined, as air will tend to accumulate there due to gravity.

Whenever the sliding sign is absent, it is crucial to locate the lung point, where the lung contacts the thoracic wall. This sign can confirm the diagnosis of pneumothorax and provide an estimate of the extent of air in the pleural space by mapping its boundaries [25].

Despite published evidence [25,26,27,28], studies using the same diagnostic criteria as in human medicine are still lacking to definitively confirm the diagnostic capabilities of T-POCUS for pneumothorax [12,29]. However, some studies [1,2] suggest that T-POCUS has higher sensitivity and specificity than traditional radiology for diagnosing pneumothorax.

Another sign of pneumothorax, described for the first time in dogs, is the abnormal curtain sign [30]. With air accumulating in the pleural space between the lung, diaphragm, and abdomen, the curtain movement of the sliding sign becomes asynchronous with respiratory cycles; thoracic and abdominal movements go in opposite directions. Also described is the double curtain sign, thought to occur when air becomes trapped in the costophrenic recess, creating a visual effect where the diaphragm contacts the thoracic wall. This sign consists of two vertical borders visible within the same ultrasound field. These borders enclose a space containing abdominal structures (such as the diaphragm), seen between two curtains of air, one cranial and one caudal. During inspiration, these two curtains separate from each other, while during expiration, they move closer together [30].

#### 3.1.2. Pleural Effusion

Regardless of the underlying pathophysiological mechanisms leading to fluid accumulation in the pleural space, pleural effusion can be classified into several types: transudate, exudate, chyle, and blood [31]. The ultrasound appearance of these types varies based on the cellular/debris content in the fluid. Transudate typically appears as an anechoic, homogeneous fluid; exudate, on the other hand, exhibits higher echogenicity and a particulate, flocculent appearance due to the presence of numerous cellular debris and inflammatory cells [32]. Additionally, any inflammatory process affecting the area will induce inflammation of the pleura itself, manifesting as increased echogenicity, thickening and irregularity of the pleura, and the appearance of fibrin strands that move with respiratory motion [33]. Chyle and blood have a similar appearance to transudate but often display echogenic particles in suspension and deposit on the visceral pleura [33,34].

It is important to note that the ultrasound appearance of pleural effusion is not always consistent with these descriptions and may occasionally present atypically. For example, the appearance of a purulent effusion can sometimes appear anechoic [35] but also could appear as soft tissue echogenicity [36]. Therefore, identifying a pleural effusion and reaching a diagnostic conclusion without prior thoracocentesis and cytological examination carries risks.

When a large volume of fluid is present in the pleural space, the pressure exerted on the lung parenchyma can overcome its resistance, causing partial or complete lung collapse and a subsequent lack of aeration. The resulting ultrasound finding is known as the jellyfish sign, in which the collapsed lung moves with a swimming motion in the fluid during respiration [32,37]. This sign suggests that the fluid has low viscosity, likely indicating transudate, as opposed to exudates, which are more viscous and thus inhibit the movement of collapsed lung tissue [38].

In a study involving trauma patients where thoracocentesis was used as a reference in the absence of CT scans, T-POCUS showed a 94% positive concordance rate and a 100% negative concordance rate [2]. These results align closely with findings reported in human medicine [9].

Pleural effusion is not universally present in cases of pneumonia or neoplastic conditions; however, it may sometimes be accompanied by other signs such as pleural line irregularity, consolidations, or an increased number of B-lines [9,39]. To optimize detection, it is advisable to search for pleural effusion in the most dependent regions, where fluids are likely to accumulate due to gravity, and to use a linear probe to enhance visualization of details.

Although formulas have been published for estimating the volume of pleural effusion in humans [40], no such formulas are available for dogs and cats. Instead, a subjective visual assessment is performed, categorizing the effusion as mild, moderate, or severe based on quantity [41]. This assessment is useful for monitoring the evolution of the effusion but remains a subjective estimate.

#### 3.1.3. Neoplastic Pleural Diseases

Due to space constraints, the reader is referred elsewhere for a detailed description of the various pleural neoplasms in dogs and cats. The identification of immobile primary pleural neoplasms can be challenging, as can certain infiltrative forms. For instance, mesothelioma lacks pathognomonic characteristics and is often associated with pleural effusion, pleural line irregularities, and small subpleural consolidations (known as the shred sign, discussed below) [42,43].

#### 3.1.4. Irregularities of the Pleural Surface

**Figure 6:**
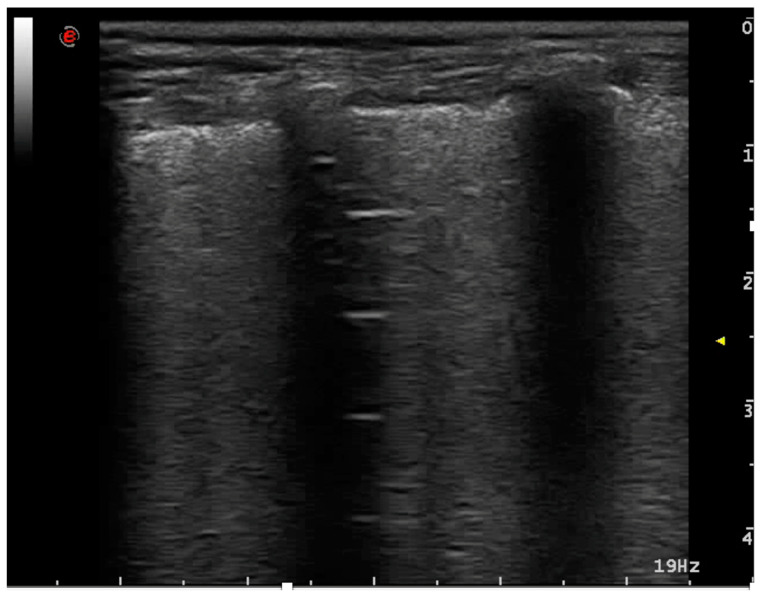
Irregular thickened pleural line with vertical artifacts.

When inflammation is the predominant etiological factor, the pleural surface becomes inflamed and adopts an appearance distinct from its normal state. It becomes irregular, rough, and thickened, which may lead to the appearance of additional signs depending on the underlying cause [44] (Figure 6). Inflammatory conditions can produce vertical artifacts originating from the pleural line (B-lines) or just below it from bronchial origins, indicating fluid accumulation. In more severe cases, this results in localized loss of aeration, manifested as the shred sign [45].

### 3.2. Pulmonary Diseases

Contrary to previous beliefs [46,47], T-POCUS can provide valuable information in diagnosing and managing various respiratory clinical scenarios. The following sections will explore the ultrasound characteristics of pneumonia, atelectasis, cardiogenic and non-cardiogenic pulmonary edema, lung lobe torsion, pulmonary fibrosis, pulmonary thromboembolism, pulmonary neoplasms, and pulmonary hemorrhage.

In many of these conditions, there is an increase in lung density as the disease progresses due to a rise in fluid/solid components within the lung, leading to a gradual loss of air and creating a characteristic tissue-like, or consolidated, appearance. In these cases, the air or gas within the bronchi becomes visible due to the contrast with the surrounding hypoechoic hepatized tissue [48]. These bronchial structures may be static, remaining immobile during the respiratory phases, or may move proximally and distally with inspiration and expiration, respectively, imparting a dynamic appearance.

#### 3.2.1. Pneumonia and Atelectasis

**Figure 7:**
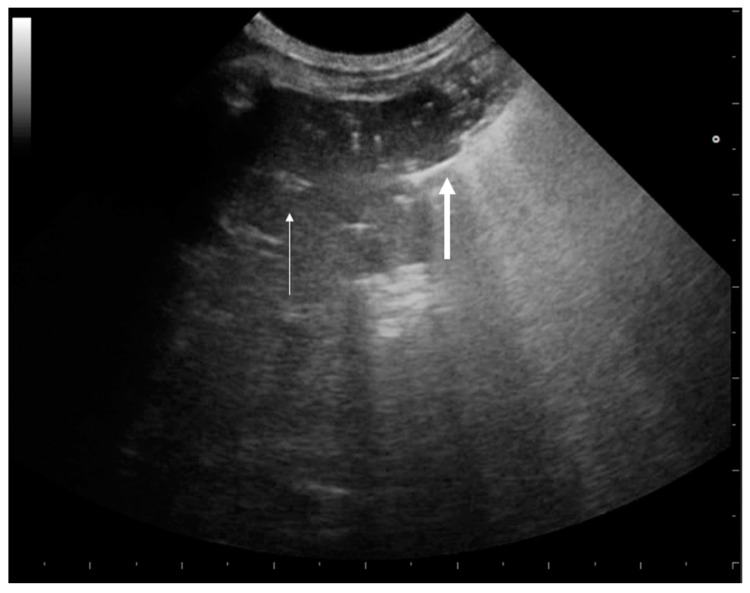
Large, consolidated area. On the left side of the image (thin white arrow), there is no aeration. On the right side (thick white arrow), the air bronchogram shows the characteristic punctate hyperechoic look as well as many vertical bronchial vertical artifacts (C-lines).

In T-POCUS, pneumonia is typically characterized by an increase in B-lines, irregularity of the pleural line, and the presence of the shred sign [7,49,50]. The absence of tissue-like signs and nodular lesions is another hallmark of pneumonia [39]. Depending on the severity of the inflammatory process, consolidations may vary in size, affecting either large portions of the lungs or smaller regions. In these cases, subpleural irregularities with small hypoechoic areas are often observed, frequently accompanied by small, static, punctate hyperechoic bronchograms [45] (Figure 7).

A common form of pneumonia in dogs is aspiration pneumonia, often due to the inhalation of foreign material. Recently, the ultrasound features of this condition have been described both at diagnosis and over time, with attention to the distribution of lesions. This form of pneumonia typically presents with an increase in B-lines, the presence of the shred sign with or without air bronchograms [51]. The lesions tend to be distributed primarily in the ventral intercostal spaces of the right hemithorax and, to a lesser degree, in the left hemithorax, due to gravity dependence [51,52].

Pneumonia and atelectasis share certain ultrasound characteristics. Both may exhibit a tissue-like appearance to varying extents, as well as air bronchograms. The air bronchogram pattern, however, can provide additional diagnostic information: a static air bronchogram may be observed in both conditions, while a dynamic air bronchogram is considered specific to pneumonia [53] (Figure 7).

Doppler imaging is another useful tool for distinguishing between the two. In atelectasis, vascular flow is typically absent, while it is present in cases of pneumonia [53,54]. More studies in dogs and cats are needed to confirm the clinical utility of this distinction, as respiratory distress is often associated with tachypnea/polypnea, leading to motion artifacts.

#### 3.2.2. Cardiogenic Pulmonary Edema

The extravascular fluid accumulation in the interlobular septa is visualized on ultrasound as an increase in B-lines with normally thickened and echogenic pleura, typically distributed homogeneously across both hemithoraces, particularly in the early stages [7,13,45,55,56].

As congestion worsens, subpleural thickening and subsequent fibrosis are observed [57], with B-lines merging into a typical coalescent pattern, eventually progressing to a fully fused and, in more severe cases, an intensely hyperechoic appearance known as white lung [58]. This occurs due to fluid accumulation in the interlobular septa, leading to thickening and congestion.

The mere presence of increased B-lines, however, is insufficient for diagnosing cardiogenic pulmonary edema [59]. To attribute this finding to a cardiac origin, a cardiac scan is essential to assess the left atrial size. This is achieved via a right parasternal short-axis view with the probe oriented toward the cardiac base. An LA/Ao ratio ≥ 2 is considered indicative of an enlarged left atrium, suggesting possible congestive heart failure [60,61]. It is also important to note that B-lines can also result from interlobular septal thickening and pulmonary fibrosis [62,63].

Due to its portability and ease of use in intensive care settings, T-POCUS can be effectively employed to monitor lesion progression over time in response to diuretic therapy.

#### 3.2.3. Non-Cardiogenic Pulmonary Edema

In the absence of left atrial enlargement, non-cardiogenic pulmonary edema is characterized by an increased number of B-lines with a non-homogeneous distribution, along with pleural line irregularities, reduced or absent sliding sign, the presence of spared areas, and tissue-like or shred signs [64]. Although published data in dogs and cats are still limited [65], the ultrasound features are comparable to those widely reported and described in human medicine [66].

#### 3.2.4. Lung Lobe Torsion

**Figure 8:**
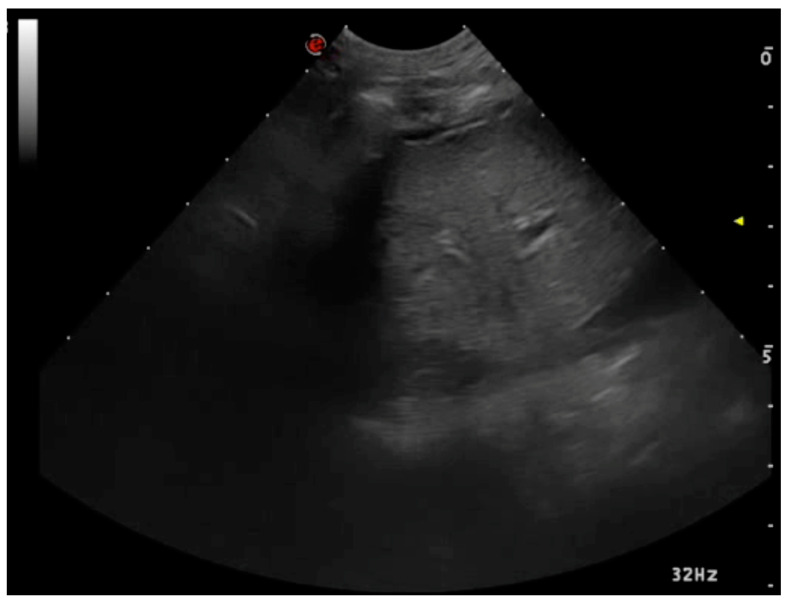
Lung lobe torsion. With an apex in depth, the torsion-affected lobe resembles a triangle. There is a pleural effusion and the lobe appears atelectatic.

In cases of lung lobe torsion in dogs and cats, the typical ultrasound findings include the tissue-like sign and pleural effusion [67,68,69]. Two additional signs have been reported, as follows: the absence of vascular flow in the twisted lung segment [66], and the hypoechoic appearance filled with scattered reverberating foci at the periphery [70]. The presence of these signs is probably affected by the period between when the lobe torsion happened and when it is examined, therefore if they are not present, the disease cannot be ruled out. Finally, motion artifacts make image interpretation and Doppler appearance more challenging (Figure 8).

Published data remain limited, and ultrasound findings should always be correlated with patient history, clinical evaluation, and, if available, CT imaging [69].

#### 3.2.5. Pulmonary Thromboembolism

Diagnosing pulmonary thromboembolism is particularly complex due to its multifactorial origin [71]. Pleuropulmonary ultrasound findings typically include subpleural consolidations with a wedge-shaped or rounded appearance, known as the wedge sign, with or without concurrent pleural effusion [72]. This appearance is due to small subpleural infarcts that create the typical ultrasound image. These findings should always be correlated with specific cardiac alterations, such as increased right ventricular afterload, right-sided chamber enlargement, abnormal interventricular septal motion, reduced left ventricular filling, distended inferior vena cava, and reduced right ventricular systolic function [73].

In dogs, the literature data are scarce; the wedge sign was reported in a case series [73], but in another case report [74], it was absent, with a more extensive tissue-like appearance observed instead.

#### 3.2.6. Pulmonary Fibrosis

Ultrasound signs of pulmonary fibrosis described in humans include an increased number of vertical artifacts [75], irregularity [76] and thickening of the pleural line [39,77], presence of subpleural cysts and small nodules [39], and reduced or absent sliding sign [76]. These signs are attributed to collagen accumulation in the subpleural interlobular septa [57].

Currently, no studies on the ultrasound characteristics of pulmonary fibrosis in dogs and cats have been published. Given that West Highland White Terriers are predisposed to fibrosis, future studies are anticipated to characterize this pathology by T-POCUS.

#### 3.2.7. Pulmonary Neoplasia

**Figure 9:**
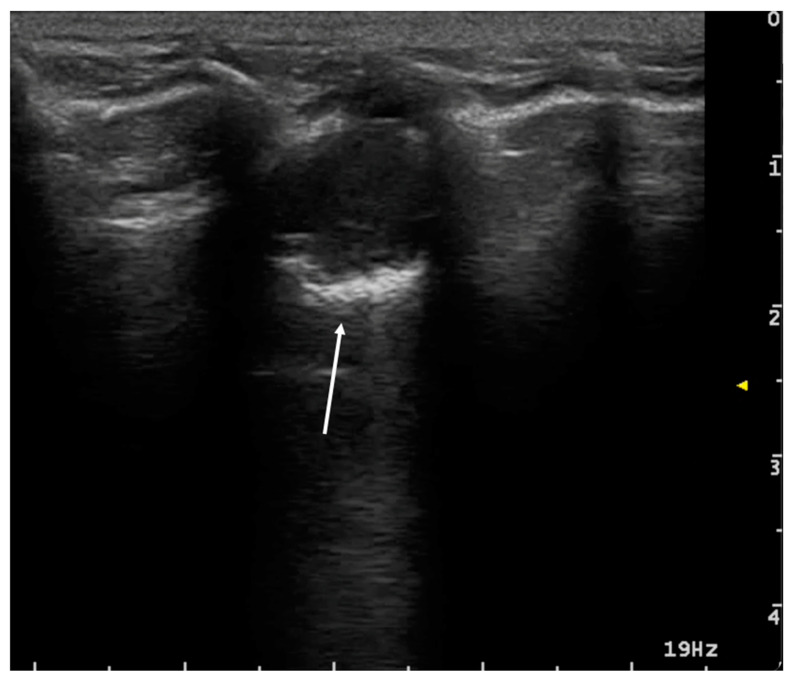
Lung nodule. Notice the typical hypoechoic rounded aspect (white arrow).

Ultrasound findings associated with various types of pulmonary neoplasms include the presence of nodules or mass-like lesions [78] (Figure 9), tissue-like signs with heterogeneous echotexture [78], subpleural thickening, and pleural effusion [79]. Enlarged mediastinal lymph nodes with heterogeneous, mixed echogenicity may also be observed (unpublished data).

Ultrasound imaging performed by positioning the probe caudally to the axilla in the ventral intercostal spaces facilitates detection. Although a study reported unsatisfactory sensitivity (60%) and specificity (65%) for detecting neoplastic nodules compared to CT [80], recent research suggests that wider scanning protocols can yield results comparable to CT [11].

Diagnostic accuracy reaches 80% when nodules, tissue-like signs, and small subpleural consolidations are diffusely present in at least three of the four thoracic quadrants [80].

Contrast-enhanced ultrasound (CEUS) is beneficial for further characterizing suspected neoplastic lesions and guiding ultrasound-guided fine-needle aspiration (FNA) procedures [81].

#### 3.2.8. Pulmonary Hemorrhage

Ultrasound signs associated with pulmonary hemorrhage include the tissue-like sign with air bronchograms due to blood, the shred sign from disrupted lung tissue, pleural effusion, pleural line irregularity with disappearance of A-lines, increased vertical artifacts due to fluid in the interstitial space, and fibrin deposits [82,83,84].

### 3.3. Miscellaneous

Various other thoracic pathologies can be identified using TPOCUS, including diaphragmatic hernias [85], postoperative adhesions [86], and the recently reported double lung point sign in dogs [11]. Conditions such as pneumomediastinum, diaphragm assessment, and bronchiectasis have not yet been described in small animals. For further insights, reference is made to the human medical literature, as current data for our species are lacking.

Additionally, a practical tool for ICU patient management involves lung ultrasound scoring systems that assist clinicians in characterizing and monitoring disease progression over time [48,87].

**Figure 10:**
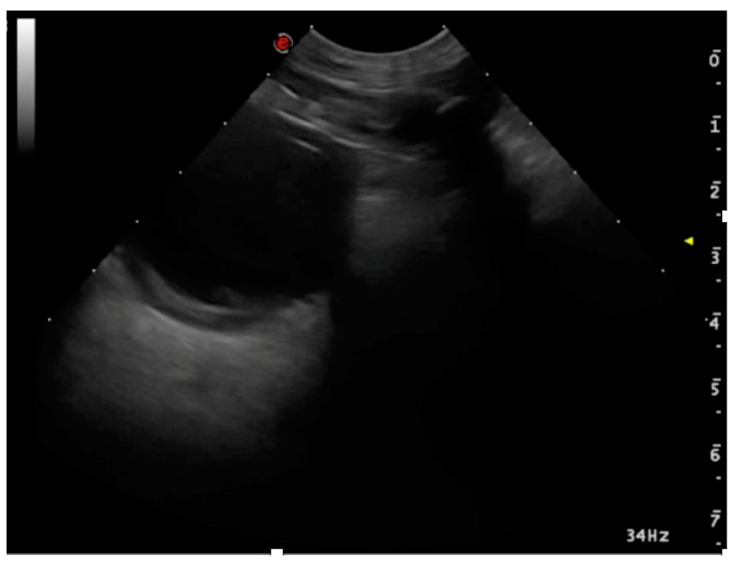
Aelurostrongylus abstrusus lung cyst. There is a circular structure with an anechoic core and an isoechoic wall.

Furthermore, pulmonary abscesses appear as rounded, isoechoic, encapsulated areas with a hypoechoic core [87]. Although they have not been documented in small animals, similar formations can also be generated by foreign materials and bronchopulmonary parasites (Figure 10).

## 4. Limitations and Future Directions

Several limitations impact the use of T-POCUS in managing critically ill small animals. First, ultrasound serves as a complementary diagnostic tool rather than a standalone replacement for clinical assessment. It enhances diagnostic accuracy when integrated into a structured approach. For instance, in a trauma patient with suspected pneumothorax, while clinical suspicion may justify thoracocentesis, T-POCUS offers a rapid, non-invasive, cost-effective, and reproducible diagnostic confirmation. Nonetheless, it is critical to recognize that T-POCUS alone may not always accurately describe the underlying illness. In certain cases, further tests, such as radiographs or advanced imaging, may be required to figure out the cause, assess the severity, or detect concurrent diseases, resulting in a more comprehensive diagnostic evaluation.

Another consideration is that thoracic changes evolve over time in accordance with the clinical course. As a result, long-term monitoring becomes critical, which complements clinical surveillance of the patient, particularly if vital indicators vary. As a result, it is beneficial to employ thoracic POCUS sheets that record the discovered lesions and their locations to assess their progression.

Like other imaging techniques, T-POCUS is operator-dependent, relying not only on practical skills but, more importantly, on theoretical knowledge. Simply reading scientific articles and handling a probe does not ensure proficiency. Proper machine settings, comprehensive understanding of artifacts, and precise scanning technique are essential pillars of effective T-POCUS.

This highlights a global issue: although many perform T-POCUS, how many have received adequate training? What should such training entail? How many hours are necessary to determine readiness for performing T-POCUS?

To address this gap in veterinary medicine—unlike recent advancements in human medicine—an interest group, VECCUS (Veterinary Emergency and Critical Care Ultrasonography), was established. This group is working toward the standardization of terminology and methodology, and it is anticipated that in the near future, VECCUS will also tackle educational aspects, with a focus on standardized training and possibly certification programs to establish proficiency benchmarks.

## 5. Conclusions

Point-of-care thoracic ultrasound in dogs and cats is experiencing rapid growth in published research, following trends seen in human medicine, albeit with a delay of approximately ten years. Many ultrasound findings align with those described in human patients, but significant progress remains to be made. Researchers worldwide are working not only to deepen the understanding of these findings, clinical indications, and implications but also to standardize protocols, establish a shared terminology, and develop specific educational pathways for essential knowledge in this field.

## Data Availability

No new data were created or analyzed in this study.
